# Effect of pulsed electric field treatment on shelf life and nutritional value of apple juice

**DOI:** 10.1007/s13197-019-03581-4

**Published:** 2019-02-07

**Authors:** Kinga Dziadek, Aneta Kopeć, Tomasz Dróżdż, Paweł Kiełbasa, Marek Ostafin, Karol Bulski, Maciej Oziembłowski

**Affiliations:** 1 Department of Human Nutrition, Faculty of Food Technology, University of Agriculture in Krakow, 122 Balicka St., 30-149 Krakow, Poland; 2 Institute of Machinery Management, Ergonomics and Production Processes, Faculty of Production and Power Engineering, University of Agriculture in Krakow, 116B Balicka St., 30-149 Krakow, Poland; 3 Department of Microbiology, Faculty of Agriculture and Economics, University of Agriculture in Krakow, 21 Mickiewicza Ave., 31-120 Krakow, Poland; 4 Department of Animal Products Technology and Quality Management, Faculty of Biotechnology and Food Sciences, Wroclaw University of Environmental and Life Science, 37 Chełmońskiego St., 51-630 Wroclaw, Poland

**Keywords:** Pulsed electric fields, Apple juice, Polyphenols, Antioxidant activity, Microorganisms

## Abstract

The aim of this study was to assess shelf life and nutritional value of apple juice, including the content of bioactive compounds, after pulsed electric field (PEF) treatment, taking into account different number of cycles: 4, 6, 8 (total 200, 300, and 400 pulses, respectively). Determination of vitamin C and polyphenols concentration, antioxidant activity as well as microbiological analysis were conducted immediately after PEF process and after 24, 48 and 72 h of storage. The results showed that PEF did not affect the content of bioactive compounds. PEF-treated juice did not show changes in the amount of vitamin C and total polyphenols during the storage for 72 h under refrigeration. PEF treatment was effective method for inactivation of a wide range of most common food spoilage microorganisms. PEF process can be used as an effective method of food preservation, allowing prolongation of shelf life and protection of nutritional value. This brings new opportunities for obtaining safe, healthy and nutritious food.

## Introduction

In recent years, there has been increasing interest in the use of various parts of electromagnetic spectrum in food processing. One of the most promising technology in food industry is pulsed electric field (PEF). During this process, the food sample is treated with high intensity electric field pulses for a short time (microseconds) in a processing chamber (Zeng et al. 2016). Depending on electric conditions, such as electric field strength and number of pulses, PEF treatment can provide different effects and can be used for various applications (Yu et al. 2015).

The PEF technology is usually used in processing liquid products, such as fruit juices, dairy products, liquid eggs and alcoholic beverages, due to ions contained in the liquid, which act as electric charge carriers. This technology is used in various food processes, such as food dehydration, sterilization, extraction, reduction of pesticide residues and inactivation of enzymes (Faridnia et al. 2015; Korma et al. 2016; Yang et al. 2016).

One of the main problem in food industry, especially when it comes to raw materials and semi-finished products, is relatively short shelf life which depends on microbiological stability and natural decay (chemical and physical processes). Spoilage caused by microorganisms is due to the presence of bacteria, yeasts and fungi which use food as a source of carbon and energy to carry out their life processes. Chemical and physical factors, responsible for food spoilage, include enzymatic and non-enzymatic reactions, temperature, moisture loss and other chemical changes affecting colour, taste and texture of food (Rawat 2015; Geveke et al. 2015).

Previous results have indicated that the PEF treatment can protect the food products against microbiological spoilage as well as the above-mentioned chemical and physical processes (Evrendilek et al. 2000; Geveke et al. 2015). Additionally the use of PEF technology can preserve or even increase the content of bioactive compounds, mainly polyphenols, increasing the nutritional value and attractiveness of the food products (Min et al. 2003b; Odriozola-Serrano et al. 2009; Medina-Meza and Barbosa-Cánovas 2015; Leong et al. 2016).

The aim of this study was to evaluate the effect of pulsed electric field treatment on shelf life and nutritional value of apple juice, taking into account different number of pulses. The content of vitamin C and polyphenols, antioxidant activity as well as spoilage microorganisms’ growth in PEF-treated juice were determined after different storage periods.

## Materials and methods

### Material

The research material was 100% apple juice, naturally unclarified, unpasteurized and unsweetened. The juice was squeezed in a local fruit and vegetable processing company (Małopolska Voivodeship, Poland) and immediately used in analysis. The content of vitamin C, polyphenols and antioxidant activity as well as microorganisms’ growth was assessed in fresh and PEF-treated juices. Furthermore, all tests were also carried out after 24, 48, and 72 h of storage under refrigeration.

### PEF system

**Fig. 1:**
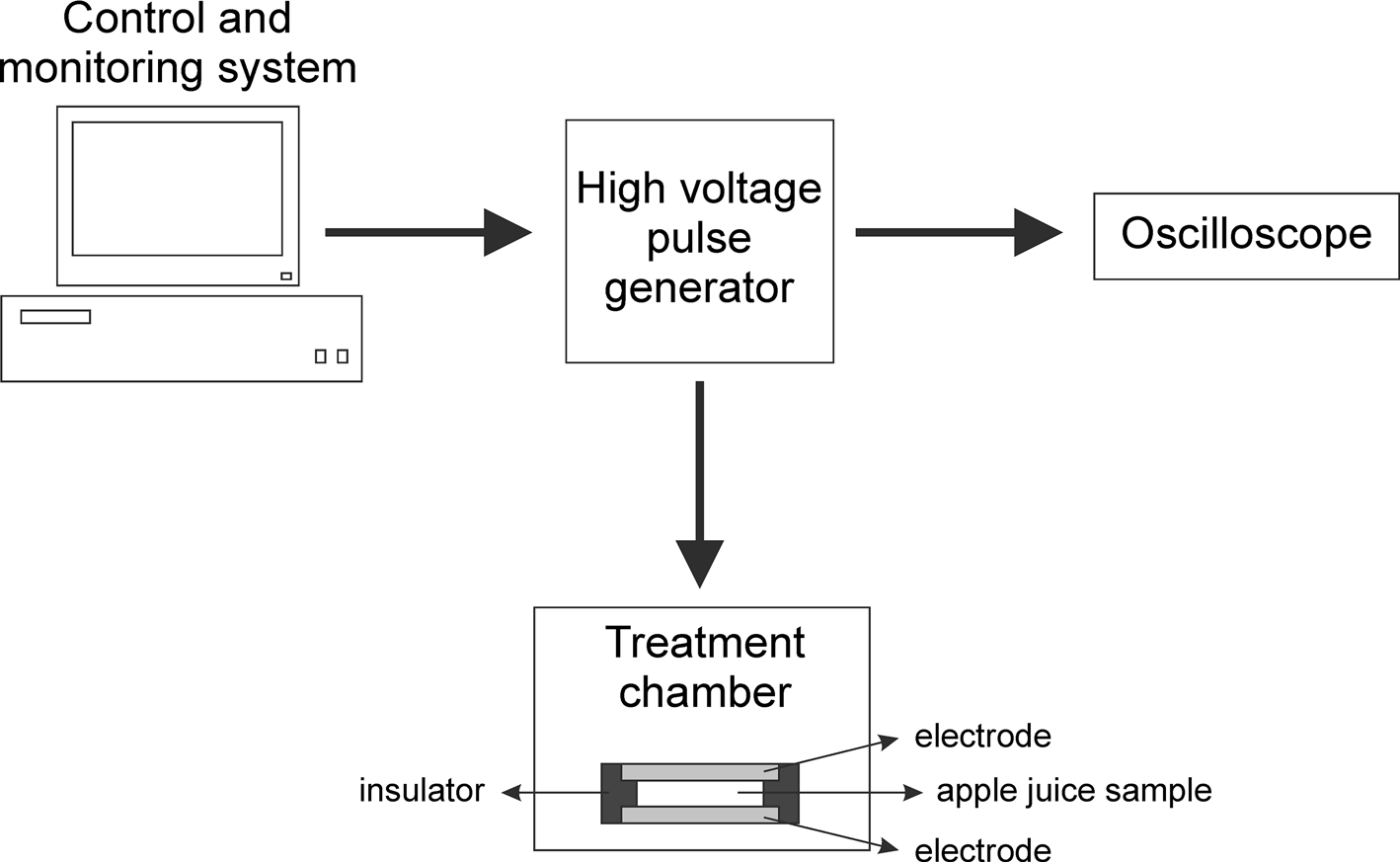
The block diagram of experimental setup

**Fig. 2:**
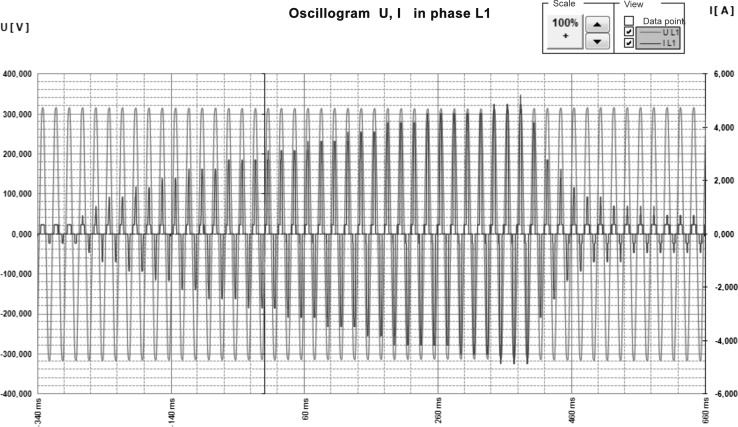
Oscillogram showing instantaneous values of voltage and current during accumulation of energy to trigger the pulse

PEF treatment was performed using a prototype PEF generator, model ERTEC-SU1 with Line Parameters Analyzer type AS3 Mini. The block diagram of experimental setup is shown in Fig. 1. During the test the analyzer was connected to the power supply line on the low-voltage side. The treatment chamber with a volume of 20 mL was made of non-conductive material. The sample was placed between the two parallel stainless steel electrodes with 10 mm distance. The analyzer was equipped with a three-phase oscilloscope to register the values of voltage and current during the accumulation of energy necessary to trigger the pulse (Fig. 2). The presented waveform indicates that the instantaneous current value, during the energy accumulation, increases from about 0.2 A to 5 A. The measurements of high-voltage pulse waveforms have shown that the voltage range was changed from 4 to 31 kV. The recorded waveforms showed oscillating changes of the pulse voltage. A pulse generated by a high-voltage generator exhibited bipolar exponential shape.

### PEF treatment

Apple juice was placed in the treatment chamber between the electrodes. The samples were subjected to cyclic PEF treatment. Each cycle consisted of 50 pulses (one pulse every 30 s). The research material was subjected to a different number of cycles: 4, 6, 8 (total 200, 300, and 400 pulses, respectively). Electric field strength was 30 kV cm^−1^. After one cycle the test sample was cooled in refrigerator at 4 °C for 15 min in order to avoid loss of vitamin C and polyphenolic compounds. Our previous study (unpublished) has indicated that used PEF treatment parameters (frequency of pulses and cycles, the total number of pulses) do not allow temperature of the sample to exceed 35 °C. The temperature was monitored before and during PEF treatment—after each 50 impulses and before the next 50 impulses (after cooling). The temperature of juice before PEF treatment was 12–13 °C, after 50 impulses—31–34 °C and after cooling—11–13 °C. Immediately after the PEF process, the juice samples were transferred to sterile tubes in order to determine the level of vitamin C, prepare methanolic extract and conduct the microbiological analysis. The PEF-treated juice was also stored for 24, 48 and 72 h.

### Determination of bioactive compounds

The total vitamin C content (ascorbic acid and dehydroascorbic acid) was measured using Tillmans Method modified by Pijanowski (Fortuna and Rożnowski 2012). The content of total polyphenols and antioxidant activity were assessed in methanolic extracts. The extracts were prepared by mixing 1 mL of juice with 40 mL of 70% methanol (POCh, Gliwice, Poland). The content of total polyphenols was estimated by the Folin-Ciocialteu reagent (Sigma-Aldrich, Saint Louis, MO, USA) (Swain and Hillis 1959). Antioxidant activity was measured by identifying the sample’s ability to extinguish an ABTS^·+^ (2, 2′-azinobis-(3-ethylbenzothiazoline-6-sulfonic acid) (Sigma-Aldrich, Saint Louis, MO, USA) free radical (Re et al. 1999).

### Microbiological analysis

The juice samples before the experiment were transferred to a sterile chamber. The chamber was sterilized in an autoclave at 121 °C for 15 min. After PEF treatment, the chamber was transferred to a laminar hood, where the samples for microbiological analyses were collected.

The research material was analyzed microbiologically using serial dilutions (Koch 1994). All dilutions were prepared using sterile buffered water (Clesceri et al. 1991). Trypic Soy Agar (Biocorp, Warsaw, Poland), Sabouraud Medium (Biocorp, Warsaw, Poland), YPD Medium (Biocorp, Warsaw, Poland), Baird Parker Agar (Sigma-Aldrich, Saint Louis, MO, USA), Stanetz and Bartley Medium (Merck, Darmstadt, Germany), Endo (Sigma-Aldrich, Saint Louis, MO, USA) and Gauze Medium were used. One milliliter of all dilutions were dispensed into the Petri plates in triplicate. The samples were incubated at 28 °C (yeasts, fungi, and actinomecetes) for 72 h and at 35 °C (bacteria) for 24 h. Plates with 30–300 CFUs per plate were counted (Koch 1994). Finally, the total count was scaled up and the cell counts were expressed as CFUs per beaker volume.

The total number of the following microorganisms were analyzed: mesophilic, psychrophilic (1) of actinomycetes on the Gauze Medium, the number of fungi on the Sabouraud Medium, yeast on the YPD Medium, coliform on the substrate Endo (2), enterococci on the Stanetz and Bartley Medium (3), the presence of *Salmonella* (4) and *Staphylococcus aureus* on Baird-Parker Medium (5). Microorganisms were grown on selective media which were sterilized in a microwave autoclave EnbioJet (Enbio Technology, Dębogórze, Poland).

### Statistical analysis

Results were expressed as the mean ± SD Differences between samples were analyzed using Duncan’s multiple range [Statistica v. 10.0 software (Tulsa, OK, USA)]. *P* values < 0.05 were regarded as significant. Correlations between the antioxidant activity and the content of bioactive compounds were examined using Pearson correlation. *P* values < 0.05 were regarded as significant.

## Results

The PEF processing, regardless of the number of pulses, did not significantly affect the content of vitamin C in apple juice (Table 1). What is more, the concentration of vitamin C in both PEF-treated and untreated juices did not change during the storage under refrigeration.

**Table 1:** The content of bioactive compounds and antioxidant activity in untreated and PEF-treated apple juice

	Number of pulses	Storage time			
		0 h	After 24 h	After 48 h	After 72 h
Vitamin C (mg 100 mL^−1^)	0	21.61 ± 2.04 a	21.61 ± 2.04 a	20.17 ± 0.01 a	21.61 ± 2.04 a
	200	23.05 ± 0.01 a	20.17 ± 4.08 a	21.61 ± 2.04 a	21.61 ± 2.04 a
	300	21.61 ± 2.04 a	21.61 ± 2.04 a	21.61 ± 2.04 a	20.17 ± 0.01 a
	400	20.17 ± 0.01 a	23.05 ± 0.01 a	21.61 ± 2.04 a	21.61 ± 2.04 a
Total polyphenols (mg 100 mL^−1^)	0	337.51 ± 29.18 e	234.83 ± 16.77 ab	232.44 ± 28.49 a	234.83 ± 21.54 ab
	200	322.39 ± 12.64 de	291.34 ± 8.27 abcde	281.79 ± 25.61 abcde	282.59 ± 11.29 abcde
	300	340.70 ± 13.58 e	272.24 ± 10.41 abcd	235.62 ± 34.80 ab	254.73 ± 13.58 abc
	400	307.26 ± 11.29 cde	300.90 ± 16.72 cde	280.88 ± 28.41 abcde	295.66 ± 22.38 bcde
ABTS^·+^ (µmol Trolox mL^−1^)	0	17.40 ± 0.45 h	12.56 ± 0.12 a	12.18 ± 0.17 a	12.50 ± 0.06 a
	200	16.17 ± 0.39 f	14.58 ± 0.13 e	14.44 ± 0.18 de	14.46 ± 0.10 de
	300	16.74 ± 0.52 g	13.97 ± 0.25 bc	13.61 ± 0.10 b	13.86 ± 0.16 bc
	400	14.09 ± 0.14 cd	19.46 ± 0.17 j	18.85 ± 0.14 i	16.69 ± 0.33 g

Results are expressed as mean ± SD (n = 3). Statistically significant different are indicated by letter code a–j (*P* < 0.05)

Furthermore, PEF treatment, regardless of the number of pulses, did not significantly influence the content of total polyphenols in apple juice (Table 1). The concentration of polyphenolic compounds did not change during the storage under refrigeration in PEF-treated juices (except the sample treated by 300 pulses), compared to untreated samples in which the content of polyphenols was reduced.

The results indicated that PEF treatment and also the number of pulses affected antioxidant activity, which decreased just after process and also after 24 h of storage (except the sample treated by 400 pulses) (Table 1).

The growth of endospores of bacteria, proteolytic bacteria, coliform, fecal streptococcus and actinomycetes in the untreated samples and samples after PEF process was not found. The highest number of mesophilic bacteria, microscopic fungi and yeast was observed in the untreated juice (Table 2). With increasing number of pulses, the reduction in number of studied microorganisms was observed. When 400 pulses were used, total inhibition of growth of mesophilic bacteria, microscopic fungi and yeasts was observed. A statistically significant differences in the number of microorganisms in the untreated sample in comparison to samples after treatment with 200, 300 and 400 pulses were found. There were no significant differences in the number of mesophilic bacteria, microscopic fungi and yeasts, between apple juice treated by 300 and 400 pulses.

**Table 2:** The average number of mesophilic bacteria, microscopic fungi and yeasts [cfu cm^−3^] in untreated and PEF-treated apple juice

Number of pulses	Mesophilic bacteria	Microscopic fungi	Yeasts
0	4450.5 × 10^4^ c	993.2 × 10^4^ c	396.9 × 10^4^ c
200	2441.3 × 10^4^ b	355.4 × 10^4^ b	221.5 × 10^4^ b
300	62 a	8.1 a	84.4 a
400	0 a	0 a	0 a

Results are expressed as mean. Statistically significant different are indicated by letter code a–c (*P* < 0.05)

**Fig. 3:**
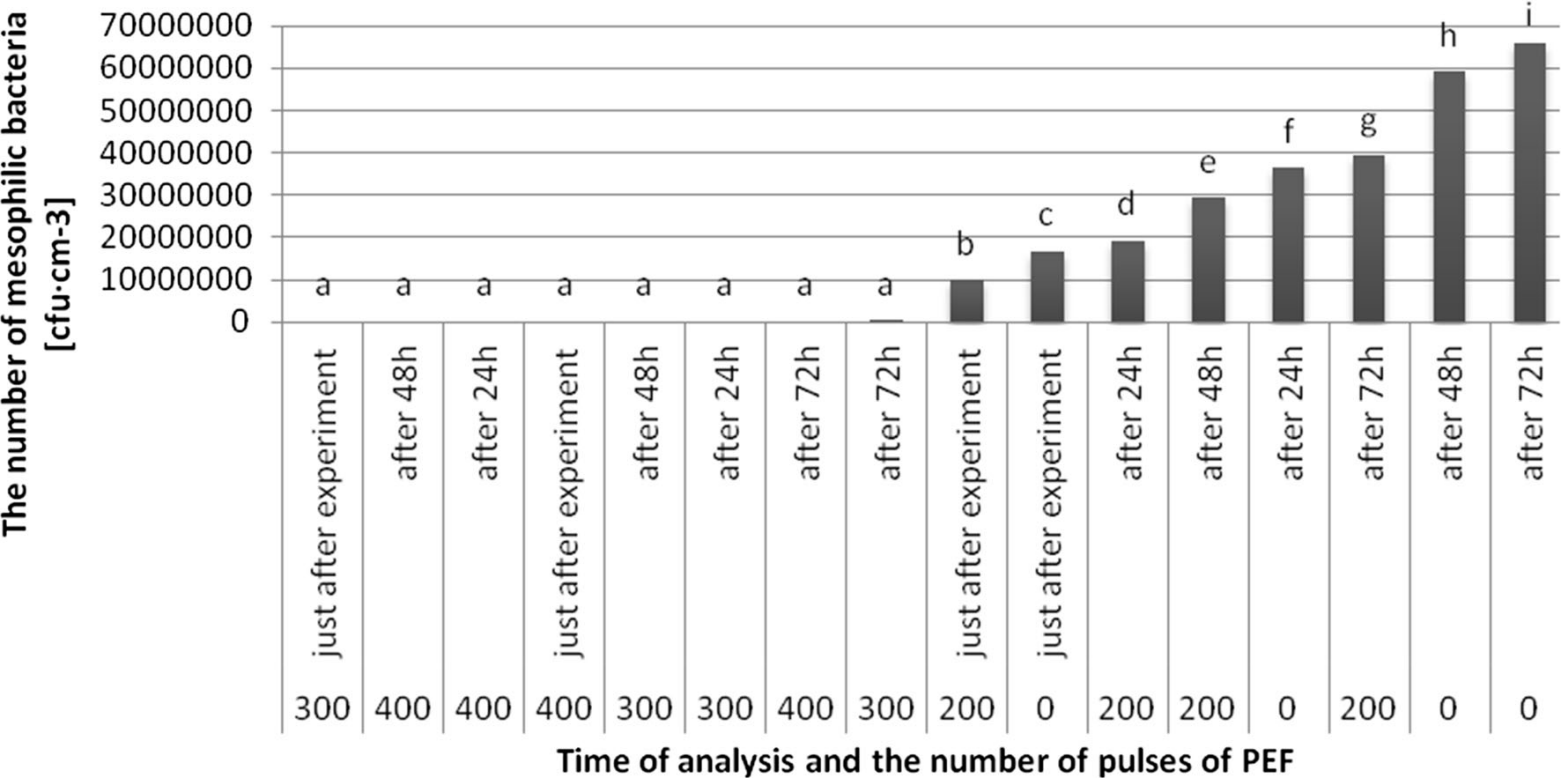
Evolution of average number of mesophilic bacteria (cfu cm^−3^) after using PEF with lapse of time after experiment

The highest average number of mesophilic bacteria was observed in the untreated juice after 72 h of storage (6586.0 × 10^4^ cfu cm^−3^), while the lowest in the juice treated with 300 pulses after 72 h (248 cfu cm^−3^) (Fig. 3). The growth of microorganisms in the juices after treatment with 300 and 400 pulses, regardless of the storage period (expect the sample treated with 300 pulses after 72 h) was not found. There were no significant differences in the number of mesophilic bacteria in the apple juice treated with 300 and 400 pulses, regardless of the storage period. The results showed significantly higher number of mesophilic bacteria in the untreated sample compared to the juice treated with 200 pulses, after the same storage periods.

**Fig. 4:**
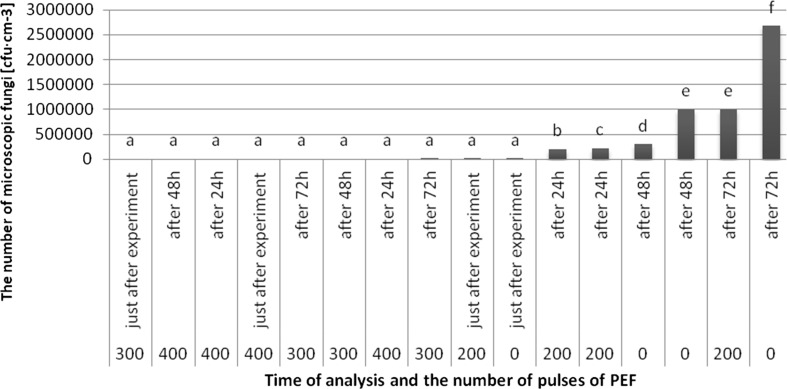
Evolution of average number of microscopic fungi (cfu cm^−3^) after using PEF with lapse of time after experiment

The highest average number of microscopic fungi was found in the untreated apple juice after 72 h of storage (267.7 × 10^4^ cfu cm^−3^), while and the lowest in juice after treatment with 300 pulses after 72 h (33 cfu cm^−3^) (Fig. 4). The growth of microorganisms in samples treated with 300 and 400 pulses, regardless of the storage period (expect the juice treated with 300 pulses after 72 h of storage) was not observed. The results showed a nonsignificant differences in the number of microscopic fungi in the apple juice after treatment with 300 and 400 pulses, regardless of the storage time. The results also revealed nonsignificant differences in the number of microscopic fungi between untreated sample and sample treated with 200 pulses just after the PEF treatment.

**Fig. 5:**
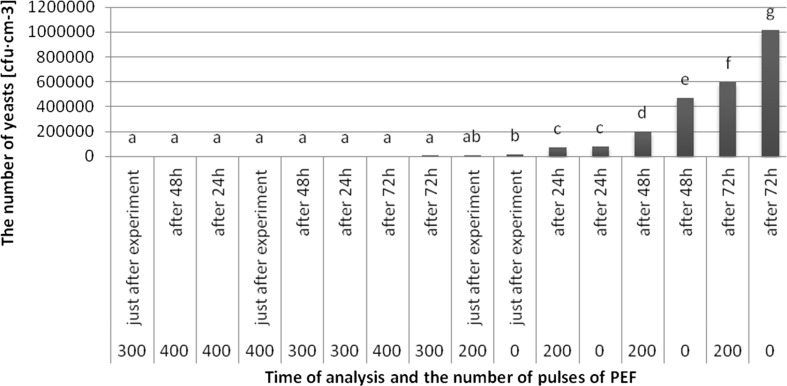
Evolution of average number of yeasts (cfu cm^−3^) after using PEF with lapse of time after experiment

The highest average number of yeasts was found in the untreated sample after 72 h (101.8 × 10^4^ cfu cm^−3^), while the lowest in juice treated with 300 pulses after 72 h (338 cfu·cm^−3^) (Fig. 5). The growth of yeasts in samples treated with 300 and 400 pulses, regardless of the storage time (expect the apple juice treated with 300 pulses after 72 h) was not observed. The obtained results indicated a nonsignificant differences in the number of yeasts in the samples after treatment with 300 and 400 pulses, regardless of the storage time. The results also showed nonsignificant differences in the number of yeasts in untreated apple juice in comparison to juice treated with 200 pulses just after the PEF treatment and after 24 h of storage. Number of yeasts in the untreated samples after 48 and 72 h of storage was higher than in the samples treated by 200 pulses after the same storage time.

## Discussion

The similar results concerning the vitamin C content directly after PEF processing were obtained by Evrendilek et al. (2000) in apple juice, Min et al. (2003a) in orange juice and Min et al. (2003b) in tomato juice. Furthermore, Evrendilek et al. (2000) reported that vitamin C content in PEF-treated apple juice did not change during storage at 4 °C and 22 °C. In contrast, the other authors showed that the content of vitamin C in the orange and orange-carrot juices decreased after storage (Min et al. 2003a; Torregrosa et al. 2006). Ascorbic acid is a heat-sensitive compound and the high temperature causes a loss of this nutrient. The lack of differences in vitamin C content between fresh and PEF-treated juice may be due to the low processing temperature (Min et al. 2003b; Elez-Martínez and Martín-Belloso 2007). Furthermore, the apple juice has a low pH in the range of 3.5–4.5. The acidic environment has shown to preserve the vitamin C (Odriozola-Serrano et al. 2009). Ascorbic acid can be stabilized by PEF processing which is related with inactivation of enzyme—ascorbate oxidase that catalyse vitamin C oxidation in fruit juices (Oms-Oliu et al. 2009).

The studies have suggested that PEF treatment may also inactivate also other enzymes, such as polyphenoloxidase and peroxidase, therefore phenolic compounds can be preserved (Bi et al. 2013; Sánchez-Vega et al. 2015). No significant differences in content of polyphenolic compounds in tomato juices just after PEF treatment were observed by Odriozola-Serrano et al. (2009). Aguilar-Rosas et al. (2007) reported that the concentration of these compounds in tested apple juice was reduced. In the literature, PEF process led to increase in the content of total polyphenols in various fruit products (plum and grape peels, grape juice) (Medina-Meza and Barbosa-Cánovas 2015; Leong et al. 2016).

Other authors have shown different results concerning the antioxidant activity. Bi et al. (2013) as well as Elez-Martínez and Martín-Belloso (2007) reported that PEF treatment did not affect the antioxidant capacity of fruit juices. The increase in antioxidant activity after PEF processing was reported by Leong et al. (2016), Oms-Oliu et al. (2009) and Odriozola-Serrano et al. (2009). Statistically significant correlation between the content of vitamin C and antioxidant activity (r = 0.3696), as well as total polyphenols and antioxidant activity (r = 0.5453) was not found. This data indicate that vitamin C and polyphenols are not the only compounds in the apple juice which are responsible for scavenging free radicals.

Inactivation of the microorganisms using PEF treatment depends on many parameters of process, i.e. field strength, length and shape of the impulse, total duration of impact, temperature and treatment time. Furthermore, selected parameters may affect each other thus it is difficult to compare the results obtained by other authors (Evrendilek and Zhang 2003). In general, the effectiveness of microorganisms inactivation increases with increasing electric field strength, temperature, treatment time and number of pulses (Buckow et al. 2013).

The literature data indicate that the main reason for cell death after PEF treatment is the formation of pores in cell membranes, known as electroporation. Mechanisms of pore formation are not clearly explained (McElhatton and Marshall 2007). One of the explanation is proposed by Zimmermann et al. (1974). During PEF process, free charges of opposite charge are accumulated on the outer and inner surfaces of cell membrane, thus the membrane is compressed. While the external electric field is higher than critical value, the pores are formed. The electric field strength and the treatment time affect pores size and their amount (Zimmermann et al. 1974; Mañas and Pagán 2005).

## Conclusion

The results showed that pulsed electric field technology did not affect the content of bioactive compounds in apple juice. What is more, PEF-treated juice did not show changes in the amount of vitamin C and total polyphenols during the storage for 72 h under refrigeration. PEF treatment of apple juice was effective method for inactivation of a wide range of most common food spoilage microorganisms, such as mesophilic bacteria, microscopic fungi and yeasts. However, there was limited number of pulses that ensure effective inactivation of these microorganisms. The use of 400 pulses allows one to store the juice for 72 h under refrigeration, while the treatment with 300 pulses ensures microbiological stability for 48 h.

To conclude, PEF process can be used as an effective method of food preservation, allowing prolongation of shelf life and protection of nutritional value. This brings new opportunities for obtaining safe, healthy and nutritious food.
